# Antimicrobial and antioxidant activities of peptide derived from turmeric plant (*Curcuma longa* L)

**DOI:** 10.1371/journal.pone.0314482

**Published:** 2024-11-25

**Authors:** Sittiruk Roytrakul, Sawanya Charoenlappanit, Suthathip Kittisenachai, Noppadon Siangpro, Jirapast Sichaem, Songkran Chuakrut, Siripun Sarin, Rumpa Jutakanoke

**Affiliations:** 1 Functional Proteomics Technology Laboratory, National Center for Genetic Engineering and Biotechnology, National Science and Technology Development Agency, 113 Thailand Science Park, Pathumthani, Thailand; 2 Office of Disease Prevention and Control, Region 3 Nakhon Sawan, Nakhon Sawan, Thailand; 3 Faculty of Science and Technology, Research Unit in Natural Products Chemistry and Bioactivities, Thammasat University Lampang Campus, Lampang, Thailand; 4 Faculty of Medical Science, Department of Microbiology and Parasitology, Naresuan University, Phitsanulok, Thailand; 5 Faculty of Medical Science, Centre of Excellence in Medical Biotechnology (CEMB), Naresuan University, Phitsanulok, Thailand; University of Tabuk, SAUDI ARABIA

## Abstract

The overuse and inappropriate use of antibiotics have led to the emergence of several antibiotic resistant bacteria. As a result, there is growing interest in exploring alternative agents as antimicrobial peptides (AMPs), which operate through unique mechanisms to effectively counteract bacterial resistance. In this study, peptides smaller than 3 kDa were isolated by cation exchange chromatography, anion exchange chromatography and reverse-phase chromatography. Subsequently, 12 candidate peptides were selected and chemically synthesized for a comparative study of growth inhibition in pathogenic bacteria. They demonstrated potent antibacterial activity toward *A*. *baumannii*, *S*. *epidermidis*, *S*. *aureus*, and *S*. *enterica*. Exposure to the Cur-1 peptide induced changes in bacterial proteins associated with metabolite interconversion and translation. In addition, all peptides derived from turmeric peptic hydrolysate exhibited antioxidant activity as assessed by ABTS, DPPH and FRAP assays. Cur-1 peptide displayed both high antibacterial and antioxidant potential, positioning it as a promising natural option for antibacterial management and applications within food industry.

## Introduction

Infectious diseases caused by pathogenic bacteria are another major problem worldwide. Synthetic antibacterial drugs are not only insufficient and expensive but also have side effects. The misuse of antibiotics gives rise to antibiotic resistance which is a serious problem in the 21^st^ Century [1]. In addition, World Health Organization (WHO) reported that approximately 200,000 deaths and 19 million disability adjusted life years (DALYs) lost annually by infectious diseases [2]. Peptides are an alternative for the treatment of infections which have many advantages, such as high efficiency of pathogenic bacteria inhibition with lower rate of antibiotic resistance and the ability to stimulate the immune systems Therefore, antimicrobial peptides (AMPs) have gained attentions to replace or be used in combination with other antibiotics [3].

Many antimicrobial peptides against pathogenic microorganisms were found to produce from microorganism [4–6]. However, antimicrobial peptides have also been found in many plant species [7]. They show specificity towards Gram-negative or Gram-positive bacteria, but most of them have broad-spectrum antibacterial activities. Antimicrobial peptides from poke weed, *Phytolacca americana* [8] and from kernels of *Macadamia integrifolia* [9], can inhibit the growth of Gram-positive bacteria. Brucin, an antibacterial peptide derived from fruit protein of a Thai medicinal plant *Brucea javanica* (L.) Merr showed a broad-spectrum antimicrobial activity against various Gram-positive and Gram-negative bacteria [10].

The turmeric plant (*Curcuma longa* L) has been recognized as a traditional Thai herbal remedy for an extended period. Its versatile applications include consumption as a food source, utilization in cloth dyeing, and incorporation into skincare regimens. Previous research efforts have demonstrated the inhibitory effects of soluble compounds extracted from the Turmeric plant against pathogenic bacteria, employing various solvents in the extraction process [11]. Nevertheless, the scientific literature exists on the investigation of protein hydrolysates and the isolation of purified peptides from the Turmeric plant. The exploration of peptides within this context holds significant promise, contributing substantial value to the resulting product. Moreover, these peptides may serve as prototypes or as a foundation for further development into medicinal or adjunctive therapeutic agents. The acquisition of these peptides involves exposure to gastric enzymes such as pepsin in the human stomach. This strategic design ensure the resilience of these bioactive peptides against degradation and facilitating their passage through the gastrointestinal tract for efficient absorption into the human body.

The primary objective of this study was to initially assess the inhibitory efficacy of protein hydrolysates derived from the turmeric plant against a diverse spectrum of pathogenic bacteria. Subsequently, peptides with a molecular weight less than 3 kDa were isolated and subjected to antioxidant and antimicrobial assessments. The bioactive active peptides were then subjected to chromatographic purification and their amino acid sequences were determined by LC-MS. Twenty-five peptides were subjected for chemically synthesis and further evaluated for their antimicrobial and antioxidant properties. This proposed research not only aims to elucidate the antioxidant characteristics but also holds promise for pharmaceutical applications, offering a novel approach for the control of pathogenic bacteria.

## Materials and methods

### Bacterial strains

Pathogenic microorganism composes of *Escherichia coli* (ATCC 25922), *Staphylococcus aureus* (ATCC 25923), *Staphylococcus epidermidis* (ATCC 12228), *Bacillus subtilis* (ATCC 6633), *Bacillus cereus* (ATCC 11778), *Pseudomonas aeruginosa* (ATCC 27853), *Vibrio parahaemolyticus* (ATCC 17802), *Klebsiella pneumoniae* (ATCC 27736), *Acinetobacter baumannii* (ATCC 19606), *Enterobacter cloacae* (ATCC 13047), *Salmonella enterica* (ATCC 13311), and *Salmonella* Typhi (ATCC 22842) were used in the experiments.

### Protein hydrolysate preparation

The rhizome of turmeric plant (*Curcuma longa* L) was finely ground in 200 mM Sodium acetate buffer at pH 4.0, followed by autoclaving at 121°C for 15 minutes. Protein concentration in the supernatant was quantified using the Lowry method [12] with Bovine Serum Albumin (BSA) as the standard. Pepsin enzyme was added at a ratio of 1:20, followed by an incubation at 37°C for 16 hours. The reaction was terminated by boiling for 10 min. The resulting mixture was filtered through a semipermeable membrane (Vivaspin 20, 3 kDa MWCO, GE Healthcare, Chicago, UK) to isolate peptides smaller than 3 kDa. The concentration of peptide was then determined by the Lowry method.

### Antimicrobial activity assay

Bacteria were initially cultured on Tryptic Soy Agar (TSA) plates at 37°C for 12–16 hours. A single colony was transferred to Tryptic Soy Broth (TSB) and incubated at 37°C with gently agitation at 100 rpm, for an additional 12–16 hours. The bacterial concentration was adjusted to an optical density at 600 nm (OD_600_) of 0.05, corresponding to 4 x 10^7^ CFU/mL, before being added to a 96-well plate (50 μL per well). Subsequently, 50 μL of protein hydrolysate or peptide was inoculated into each well at a final concentration of 100 μg/mL, and the mixture was incubated at 37°C with continuous shaking at 100 rpm for 6 hours. Wells with inoculated broth alone served as a negative control, while wells containing Ampicillin (100 μg/mL) served as a positive control. Following incubation, 25 μL of a 100 μg/mL MTT (3-(4,5-dimethylthiazol-2-yl)-2,5-diphenyltetrazolium bromide) solution was added to each well, thoroughly mixed, and incubated at room temperature for 4 hours. The optical density at 570 nm (OD_570_nm) was subsequently measured using a microplate reader (Synergy H1 Hybrid Multi-Mode Reader, Biotek, Winusky, VT, USA). The percentage of inhibition was calculated using the provided equation:

$$[(OD_{570}\;control–OD_{570}\;test)/OD_{570}\;control]\times 100$$ (1)

### Antioxidant activity assay

#### ABTS assay

Radical scavenging antioxidant activity was determined according to the ABTS radical scavenging activity following the method described by Re et al. [13] with slight modifications. Briefly, the solution of ABTS radical was prepared from the reaction between 7 mM ABTS (2,2-azino-bis (3-ethylbenzothiazoline-6-sulphonic acid) diammonium salt) and 2.45 mM ammonium persulphate. The resulting ABTS solution was diluted with distilled water to achieve an optical density of 0.7 at 750 nm. Then, a sample or standard of 10 μL was mixed with 190 μL of ABTS solution and incubated for 5 minutes in the dark. The absorbance was measured at 750 nm by a microplate reader (Synergy H1 Hybrid Multi-Mode Reader, Biotek, Winusky, VT, USA). Ascorbic acid was used as the standard. Antioxidant capacity was exhibited as μg ascorbic acid equivalent (μg AAE·g^−1^).

#### FRAP assay

The ferric ion-reducing antioxidant power assay was analyzed according to the method by Rumpf et al. [14] with minor modifications. Briefly, the FRAP reagent was prepared from 300 mM sodium acetate (pH 3.6) and 10 mM TPTZ (2,4,6-tris (2-pyridyl)-s-triazine) in 40 mM HCl and 20 mM ferric chloride at the ratio of 10:1:1, respectively. Then, a 10 μL aliquot of the sample or standard was mixed with 190 μL of the FRAP reagent before incubating in the dark for 30 minutes. The absorbance was measured at 593 nm using a microplate reader (Synergy H1 Hybrid Multi-Mode Reader, Biotek, Winusky, VT, USA). Ascorbic acid (Sigma-Aldrich, Singapore) was used as the standard. Results were expressed as micrograms of ascorbic acid equivalent per gram (μg AAE g^−1^).

### DPPH radical scavenging assay

The DPPH radical scavenging activity of the sample was evaluated following the protocol by Muangrod et al. [15] with slight modifications. Briefly, 190 μL of 0.1 mM DPPH was mixed with 10 μL of the sample or standard ascorbic acid in each well and incubated at room temperature in the dark for 30 minutes. The absorbance of the mixture was measured at 517 nm using a microplate reader (Synergy H1 Hybrid Multi-Mode Reader, Biotek, Winusky, VT, USA). The percentage inhibition of DPPH radical scavenging activity was calculated using the following equation:

$$\%DPPH\;inhibition=[(A_{control}-A_{sample})/A_{control}]\times 100$$ (2)

where A _control_ means the absorbance of the control that contained all reagents except the test samples, and A _sample_ means the absorbance of the mixture (sample and 0.1 mM DPPH). Ascorbic acid was used as the standard. Results were expressed as μg ascorbic acid equivalent (μg AAE g^−1^).

### Purification of bioactive peptides from protein hydrolysates by chromatographic technique

Protein hydrolysate with discerned antimicrobial properties was subjected to a multi-step purification process involving ion exchange chromatography (both cation and anion) and reversed phase chromatography. The protein hydrolysate was initially processed through the C18 spin column (Amberlite ^®^XAD^®^-2). The column was subsequently washed with 0.1% formic acid and sterile water to remove impurities and peptides were eluted with 100% acetonitrile. The eluent was evaporated at 50°C in a hot air oven. The dried sample was redissolved in 10 mM Sodium acetate buffer (pH 4.0). For further purification, cation exchange chromatography (HiTrap SP FF Sepharose^TM^) and anion exchange chromatography (HiTrap Q FF Sepharose^TM^) were applied, sequentially. The 1 M sodium chloride (NaCl) was used to elute the peptides from both columns. The eluent from the cation exchange was subjected to the anion column, the unbound anion exchange fraction was applied to the C18 column and stepwise eluted with varying acetonitrile concentration (20%, 40%, 60%, 80%, and 100%). Antibacterial activity of all fractions was assayed and fractions having activity were selectively collected for peptide sequencing by LC-MS. The antioxidant activity (DPPH, ABTS, FRAP) of all frication was thoroughly analyzed.

### Peptide sequencing by LC-MS

To examine peptide sequences, the bioactive peptide fraction was purified using C18 ZipTip (Merck Millipore, Darmstadt, Germany). The concentration of peptides was measured via the Bradford method [16] with bovine serum albumin as the standard. Then, peptide samples were analyzed by LC-MS on an Ultimate3000 Nano/Capillary LC System (Thermo Scientific, Loughborough, UK) linked to a Hybrid quadrupole Q-Tof impact II™ (Bruker Daltonics, MA, USA) equipped with a Nano-captive spray ion source. Specifically, 100 ng of peptide sample was concentrated on a μ-Precolumn (300 μm i.d. × 5 mm) packed with C18 Pepmap 100 (5 μm, 100 Å, Thermo Scientific, Loughborough, UK). Peptide separation was occured on a 75 μm i.d. × 15 cm column packed with Acclaim PepMap RSLC C18 resin (2 μm, 100 Å, nanoViper, Thermo Scientific, Loughborough, UK). The C18 column was maintained in a thermostatic column oven at 60°C. A gradient of solvent A (0.1% aqueous formic acid) and solvent B (0.1% formic acid in 80% acetonitrile) were used for peptide elution (5–55% solvent B for 30 min) at a flow rate of 0.3 μL/min. Electrospray ionization was performed at 1.6 kV with nitrogen as the drying gas (flow rate ~50 L/h). The collision-induced-dissociation (CID) generated product ion mass spectra using nitrogen as the collision gas. Mass spectra (MS) and MS/MS spectra were acquired in the positive-ion mode at 2 Hz over the range (m/z) of 150–2200 with collision energy adjusted to 10 eV according to the m/z value. The LC-MS analysis of each sample was done in triplicate. The Andromeda search engine was used to match MS/MS spectra to the Uniprot Zingiberaceae database, and MaxQuant 2.2.0.0 was used to quantify the peptides in individual samples.

### Peptide synthesis

The synthesis of peptide was performed by GenScript (Piscataway, NJ, USA) using Fmoc solid-phase synthesis. All peptides used had a purity of more than 95% based on mass spectrometry technique. Their physio-chemical properties were calculated using ThermoFisher Scientific Peptide Analyzing Tool for molecular weight and hydrophobicity (https://www.thermofisher.com/th/en/home/life-science/protein-biology/peptides-proteins/

custom-peptide-synthesis-services/peptide-analyzing-tool.html), INNOVAGEN Peptide property calculator (for isoelectric point and Net charge) [17]. Peptide secondary structure, helix percentage and coil percentage were obtained using PEP2D [18].

### Shotgun proteomics analysis of bacteria after exposure to Cur-1 peptide

Bacteria were incubated with 100 μg/mL Cur-1 peptide at 37°C with continuous shaking at 100 rpm for 6 hours. The cells were harvested with centrifugation and the resulting pellet was washed once with distilled water. Bacterial cells were lysed with 0.5%SDS and protein concentration was measured by Lowry assay using BSA as a standard protein [12]. Five micrograms of protein samples were reduced disulfide bonds using 5 mM dithiothreitol (DTT) in 10 mM AMBIC at 60°C for 1 hour. Alkylation of sulfhydryl groups was performed using 15 mM Iodoacetamide (IAA) in 10 mM AMBIC at room temperature for 45 minutes in the dark. The protein samples were digested with sequencing grade porcine trypsin (1:20 ratio) for 16 hours at 37°C. The resulting tryptic peptides were dried using a speed vacuum concentrator and resuspended in 0.1% formic acid for LC-MS analysis.

MaxQuant 2.2.0.0 was used to quantify the proteins in individual samples using the Andromeda search engine to correlate MS/MS spectra against the Uniprot database for *A*. *baumannii*, *S*. *aureus*, *S*. *epidermidis*, and *S*. *enterica* [19]. Label-free quantitation was performed with MaxQuant’s standard settings, which included a maximum of two missed cleavages, a mass tolerance of 0.6 dalton for the main search, and trypsin as digesting enzyme. Carbamidomethylation of cystein was set as a fixed modification, while oxidation of methionine and acetylation of the protein N-terminus were specified as variable modifications. Protein identification criterior required a minimum length of 7 amino acids and the presence of at least two peptides, including one unique peptide. Protein FDR was set at 1% estimated using the reversed search sequences. The maximal number of 5 modifications per peptide was permitted during analysis. The database for protein searches comprised bacterial proteome sequences downloaded from Uniprot (October 15, 2023).

The visualization and statistical analyses of the LC-MS data, including Principal Component Analysis (PCA), differential analysis (volcano plot, ANOVA and heatmap) was conducted using Metaboanalyst 6.0 with a significance threshold of *P*-value < 0.05 [20]. Gene ontology (GO) enrichment analysis was conducted on differentially expressed proteins using the Panther 18.0 [21].

## Results

### Antimicrobial and antioxidant activity of <3 kDa peptides derived from pepsin hydrolysate

The antibacterial activity of protein hydrolysate containing <3 kDa peptides from turmeric sample (100 μg/mL) was tested by broth dilution method. Peptic turmeric hydrolysate displayed antibacterial efficiency, with inhibitory percentages ranging from 0.15% to 31.46% against every human pathogenic bacterium (except *S*. *aureus* ATCC 25923, -3.36% and *P*. *aeruginosa* ATCC 27853, -9.32%). Among the tested pathogens, only *A*. *baumannii* ATCC 19606 exhibited high inhibition (>30%), as shown in Table 1. Turmeric hydrolysate showed strong inhibitory activity against *A*. *baumannii*, *S*. *epidermidis*, *S*. *aureus*, and *S*. *enterica*, then these bacteria were selected for monitoring the antibacterial activity of each purified fraction.

**Table 1:** Ranking of inhibitory percentage of turmeric hydrolysate containing <3 kDa peptides against human pathogenic bacterial growth at 6 h after treatment.

Antibacterial Activity Ranking	Human Pathogenic Bacteria	Inhibitory Percentage (Means±SD)	
		Turmeric hydrolysate	Ampicillin
**1**	*Acinetobacter baumannii* (ATCC 19606)	58.09±1.33	42.01±4.67
**2**	*Staphylococcus aureus* (ATCC 25923)	37.88±3.85	-2.72±0.02
**3**	*Salmonella enterica* (ATCC 13311)	17.75±0.46	84.60±4.34
**4**	*Staphylococcus epidermidis* (ATCC 12228)	15.02±0.75	7.42±0.23
**5**	*Bacillus subtilis *(ATCC 6633)	10.64±1.35	96.54±4.03
**6**	*Salmonella* Typhi (ATCC 22842)	4.80±0.20	8.60±0.22
**7**	*Pseudomonas aeruginosa *(ATCC 27853)	3.76±0.08	16.29±0.87
**8**	*Enterobacter cloacae* (ATCC 13047)	-6.69±0.26	15.64±0.89
**9**	*Escherichia coli *(ATCC 25922)	-13.96±0.08	82.10±2.62
**10**	*Klebsiella pneumoniae* (ATCC 27736)	-14.94±0.64	-19.13±1.32
**11**	*Bacillus cereus* (ATCC 11778)	-25.70±0.88	35.91±1.46
**12**	*Vibrio parahaemolyticus* (ATCC 17802)	-39.46±0.54	-45.81±1.54

In addition, the turmeric protein hydrolysate possessed DPPH and ABTS radical-scavenging activity, as well as ferric reducing antioxidant power (FRAP). At a concentration of 100 μg/mL, the <3 kDa peptides from turmeric hydrolysate effectively scavenged ABTS and DPPH free radicals, yielding 1.24±0.01 and 0.14±0.03μg AAE·g^−1^, respectively. However, no FRAP reducing power was observed.

### Purification of bioactive peptides from protein hydrolysate

The majority of antimicrobial peptides (AMPs) consisted of short chain amino acids and typically contain two or more positive charged amino acids, especially arginine and lysine. Additionally, over 50% of amino acids residues in AMPs are hydrophobic [22,23]. To enhance likelihood of obtaining cationic peptides, the turmeric hydrolysate was then initially purified by cation exchange chromatography (CEx). The CEx-bound fraction was eluted with 1 M NaCl and further purified through anion exchange chromatography (AEx) to eliminate anioic peptides. Only the AEx unbound fraction was collected and subjected to purify via stepwise elution on a hydrophobic column chromatography (C18). Peptides eluted at 60% acetonitrile (ACN) showed the highest inhibitory percentage against *A*. *baumannii*, *S*. *aureus*, *S*. *epidermidis*, and *S*. *enterica* as indicated in Table 2.

**Table 2:** Inhibitory percentage of purified fraction of turmeric hydrolysate against 4 human pathogenic bacterial growth at 6 h after treatment.

Sample	Inhibitory Percentage (Means±SD)			
	*Acinetobacter baumannii* ATCC 19606	*Salmonella* *enterica* ATCC 13311	*Staphylococcus aureus* ATCC 25923	*Staphylococcus epidermidis* ATCC 12228
Bound cation	8.41±0.34	3.40±0.05	18.84±0.59	6.42±0.05
Bound cation\Unbound anion	24.83±1.19	7.66±0.04	11.11±0.55	11.28±0.29
Bound cation\Unbound anion\Bound C18 (20% ACN)	-5.44±0.32	-2.79±0.68	7.87±0.26	34.62±2.96
Bound cation\Unbound anion\Bound C18 (40% ACN)	-3.27±0.13	1.17±0.02	15.21±0.54	4.15±0.26
Bound cation\Unbound anion\Bound C18 (60% ACN)	-1.21±0.06	4.47±0.10	30.41±0.98	9.15±0.18
Bound cation\Unbound anion\Bound C18 (80% ACN)	2.75±0.12	-0.29±0.01	-10.51±0.71	-1.30±0.08
Bound cation\Unbound anion\Bound C18 (100% ACN)	10.35±0.11	4.85±0.24	-18.95±0.91	8.43±0.32

In addition, to evaluate the antioxidant activity of peptides derived from turmeric hydrolysate, all purified fractions underwent analysis using ABTS, DPPH and FRAP assays. ABTS radical scavenging activity fell within the range of 0.28–1.27 μg AAE g^−1^. The DPPH radical scavenging activity of purified peptides ranged from 0.43 to 0.55 μg AAE g^−1^, while the FRAP of purified peptides spanned from 0 to 0.70 μg AAE g^−1^. As indicated in Table 3, the peptides fraction eluted from the C18 column using 60% ACN demonstrated high ABTS radical scavenging activity, DPPH radical scavenging activity and FRAP. Taken together, this peptide fraction, which exhibited both potent antibacterial and antioxidant activity was then selected for further analysis of their amino acid sequence by LC-MS. Peptide sequences with high Andromeda scores and matched with Zingiberaceae peptides were synthesized using a solid-phase peptide synthesis method.

**Table 3:** ABTS radical-scavenging activity, DPPH radical scavenging activity, and FRAP of turmeric crude hydrolysate, bound, and synthetic peptide.

Sample	Antioxidant activities (μg AAE g^−1^± SD)		
	ABTS	DPPH	FRAP
Bound cation	1.26±0.06	0.89±0.02	0
Bound cation\Unbound anion	1.27±0.00	0.51±0.01	0
Bound cation\Unbound anion\Bound C18 (20% ACN)	1.27±0.02	0.40±0.01	0
Bound cation\Unbound anion\Bound C18 (40% ACN)	1.27±0.12	0.44±0.01	0
Bound cation\Unbound anion\Bound C18 (60% ACN)	1.23±0.02	0.55±0.01	0.01±0.00
Bound cation\Unbound anion\Bound C18 (80% ACN)	0.56±0.00	0.46±0.00	0.67±0.12
Bound cation\Unbound anion\Bound C18 (100% ACN)	0.28±0.00	0.43±0.02	0.70±0.03

### Antimicrobial activity of synthetic peptides derived from turmeric hydrolysate

In this study, peptides in a purified fraction having potent antimicrobial and antioxidant activity were sequenced by LC-MS (Table 4). Twelve peptides with high peptide scores and fewer than fifteen amino acid residues were selected for determination of antimicrobial activity (Table 5). The resultants indicated very evidently that peptide Cur-25 (KLHLLILI) and Cur-14 (PLLLKLIV) inhibited growth of both gram positive (*S*. *epidermidis* ATCC 12228 and *S*. *aureus* ATCC 25923) and gram negative bacteria (*A*. *baumannii* ATCC 19606 and *S*. *enterica* ATCC 13311). However, they demonstrated a remarkable efficacy against *S*. *epidermidis* ATCC 12228 and *A*. *baumannii* ATCC 19606.

**Table 4:** Amino acid sequence and predicted physio-chemical properties of synthetic peptides.

Peptide	Peptide sequence	Molecular weight (Da)	pI	Net charge	Hydrophobicity (%)	Helix (%)	Coil (%)	Secondary structure
Cur-1	KLHLLILI	961.6689	9.91	1.1	38.24	37.50	62.5	CCCHHHCC
Cur-2	PLLLKLIV	907.6471	10.57	1	38.51	50	50	CCHHHHCC
Cur-3	VKLKIPLLIL	1148.8261	10.72	2	41.08	40	60	CCCCCHHHHC
Cur-4	PKVKILIL	922.6580	10.91	2	33.61	25	75	CCCCHHCC
Cur-5	LKKTLLLVNKADLLP	1678.0758	10.44	2	37.96	53.33	46.66	CCCHHHHHHHHCCCC
Cur-6	VLKKLLPLL	1035.7420	10.72	2	38.88	33.33	66.67	CCCHHHCCC
Cur-7	PLKLLLPL	905.6314	10.57	1	38.24	12.50	87.50	CCCHCCCC
Cur-8	ARLLLLKP	922.6328	11.42	2	30.04	37.50	62.5	CCHHHCCC
Cur-9	QIILLLLKP	1049.7213	9.83	1	37.96	44.44	55.56	CCHHHHCCC
Cur-10	VHVLLLIL	918.6267	7.78	0.1	41.97	50	50	CCHHHHCC
Cur-11	LKKPVILL	922.6580	10.73	2	33.11	0	100	CCCCCCCC
Cur-12	HAFRDAVTPLE	1254.6357	5.17	-0.9	25.56	0	100	CCCCCCCCCCC

**Table 5:** Inhibitory percentages of synthetic peptides derived from purified protein hydrolysates of turmeric.

	Gram-positive		Gram-negative	
Peptide Name	*S*. *aureus* ATCC 25923	*S*. *epidermidis* ATCC 12228	*A*. *baumannii* ATCC 19606	*S*. *enterica* ATCC 13311
Cur-1	11.56±0.01	28.29±0.01	29.60±0.02	17.77±0.02
Cur-2	21.23±0.03	25.23±0.01	14.88±0.01	11.94±0.02
Cur-3	33.04±0.02	18.74±0.02	7.08±0.02	-22.73±0.02
Cur-4	1.02±0.03	40.67±0.01	1.82±0.03	-14.99±0.01
Cur-5	11.25±0.02	0.76±0.01	4.47±0.03	-1.39±0.02
Cur-6	3.73±0.03	6.23±0.04	1.54±0.03	-0.14±0.04
Cur-7	1.79±0.01	1.44±0.02	1.31±0.02	-7.06±0.02
Cur-8	10.49±0.01	-0.93±0.01	12.86±0.01	7.60±0.01
Cur-9	-2.86±0.01	16.33±0.01	10.92±0.03	11.94±0.03
Cur-10	-2.30±0.03	16.16±0.02	7.08±0.03	11.70±0.01
Cur-11	5.58±0.01	-0.17±0.02	8.15±0.02	1.49±0.03
Cur-12	2.30±0.03	-2.84±0.01	10.49±0.02	0.31±0.01

### Antibacterial mechanism of Cur-1 peptide

**Fig 1:**
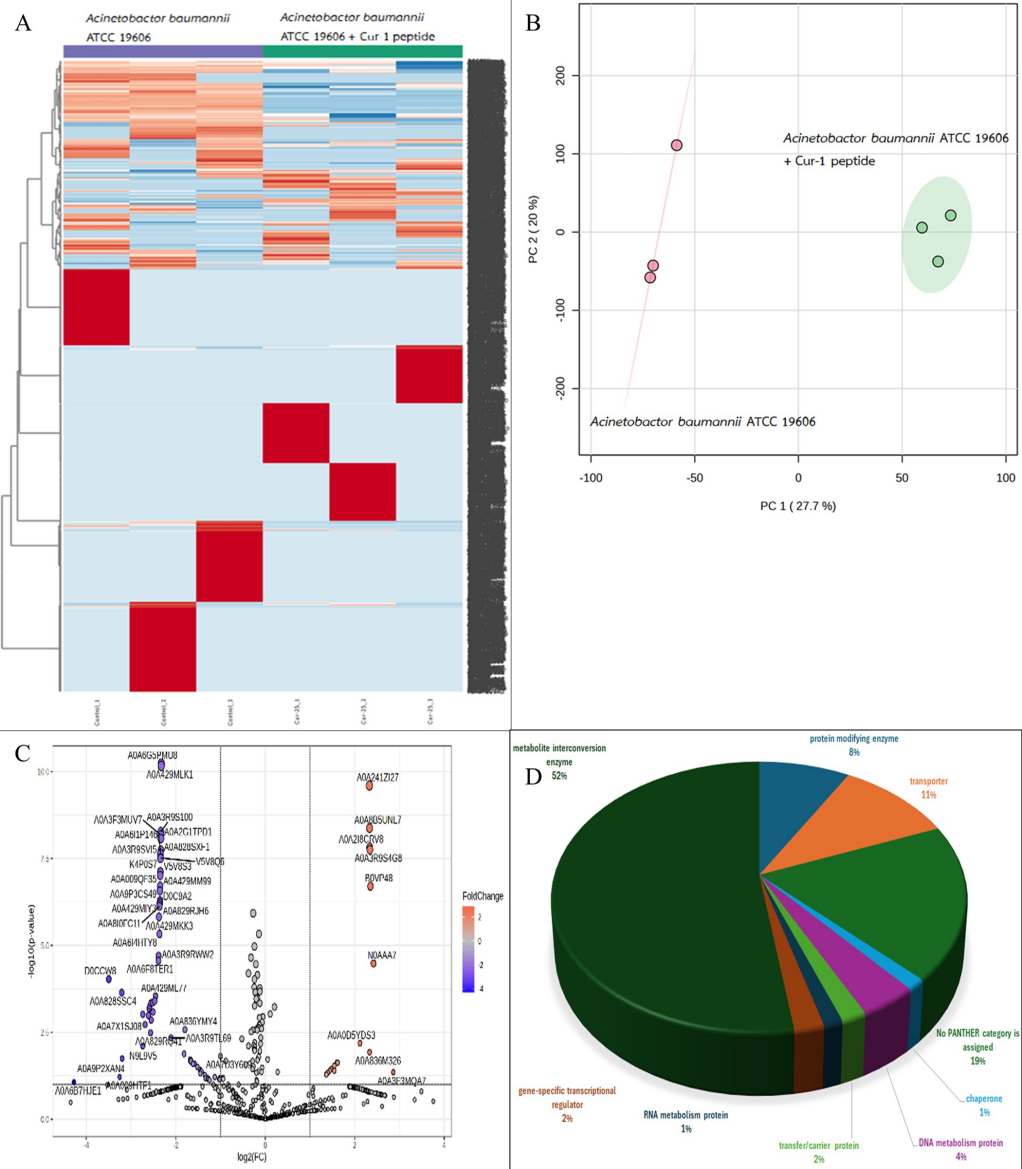
Proteomic profile of *Acinetobacter baumannii* ATCC 19606 in response to peptide cur-1. (A) Heatmap represents protein expression level. (B) Principal component analysis of protein profiles. (C) Volcano plot analysis shows the differential expressed proteins. (D) Functional analysis of the differential expressed proteins.

**Fig 2:**
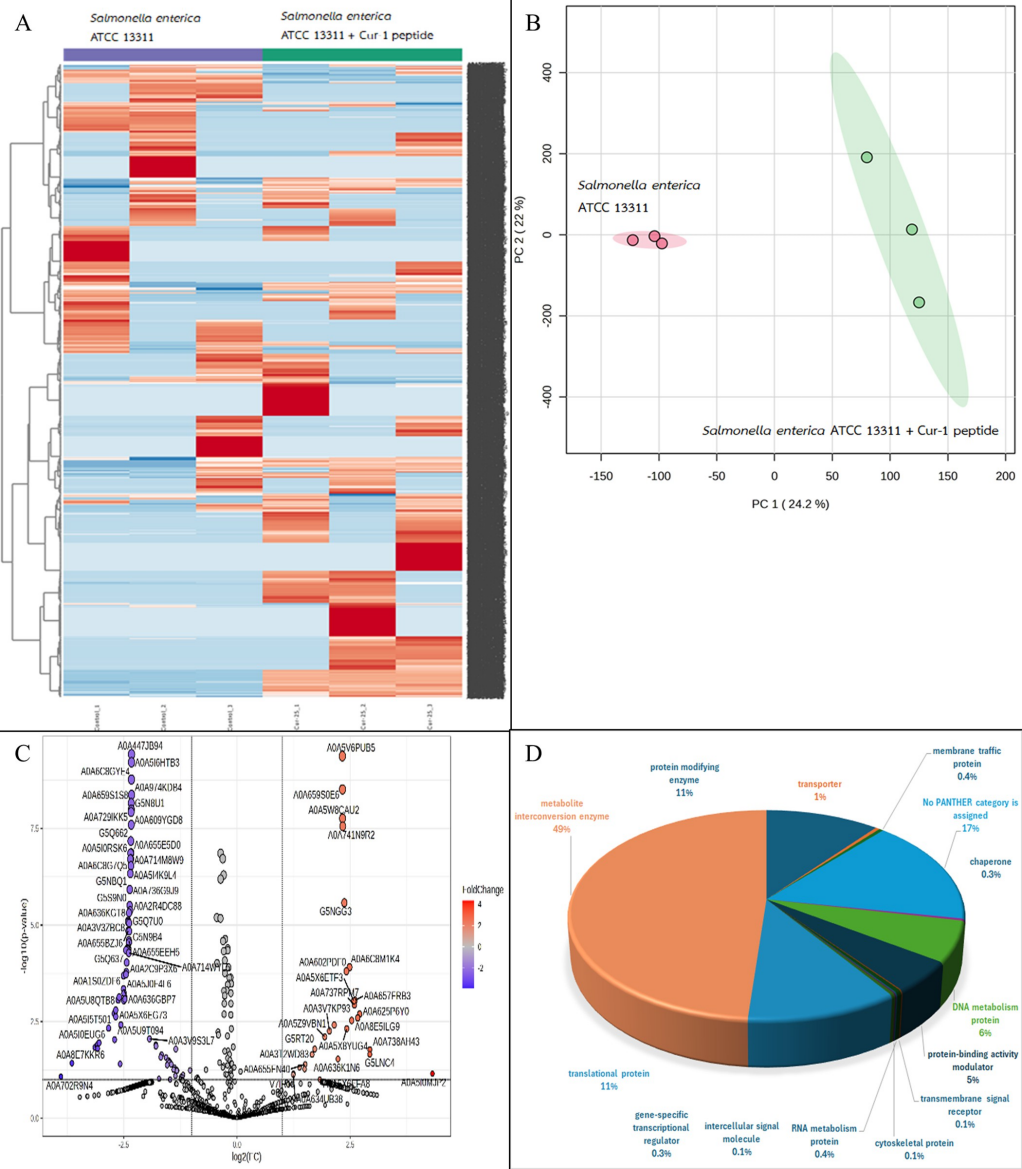
Proteomic profile of *Salmonella enterica* ATCC 13311 in response to peptide cur-1. (A) Heatmap represents protein expression level. (B) Principal component analysis of protein profiles. (C) Volcano plot analysis shows the differential expressed proteins. (D) Functional analysis of the differential expressed proteins.

**Fig 3:**
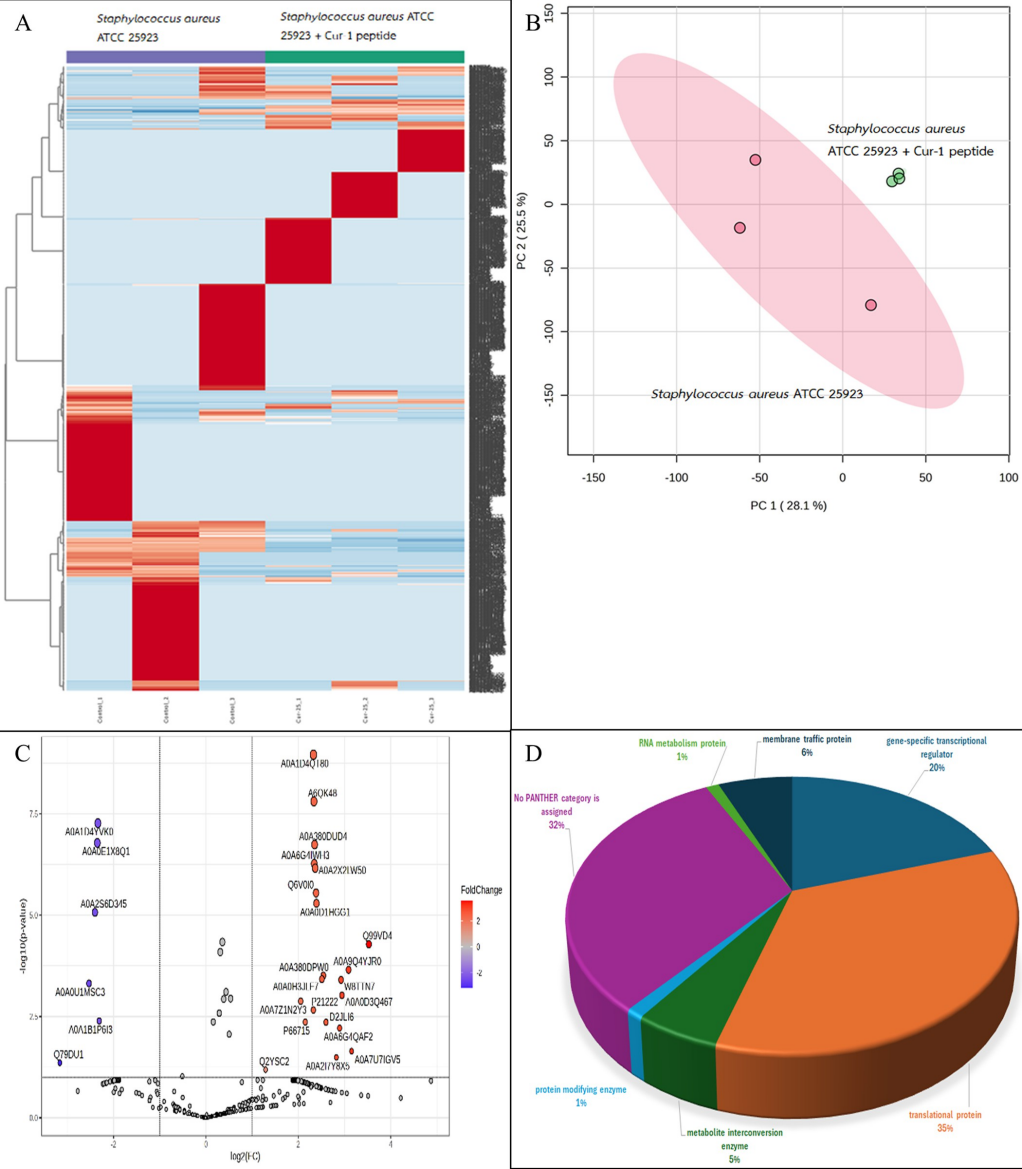
Proteomic profile of *Staphylococcus aureus* ATCC 25923 in response to peptide cur-1. (A) Heatmap represents protein expression level. (B) Principal component analysis of protein profiles. (C) Volcano plot analysis shows the differential expressed proteins. (D) Functional analysis of the differential expressed proteins.

**Fig 4:**
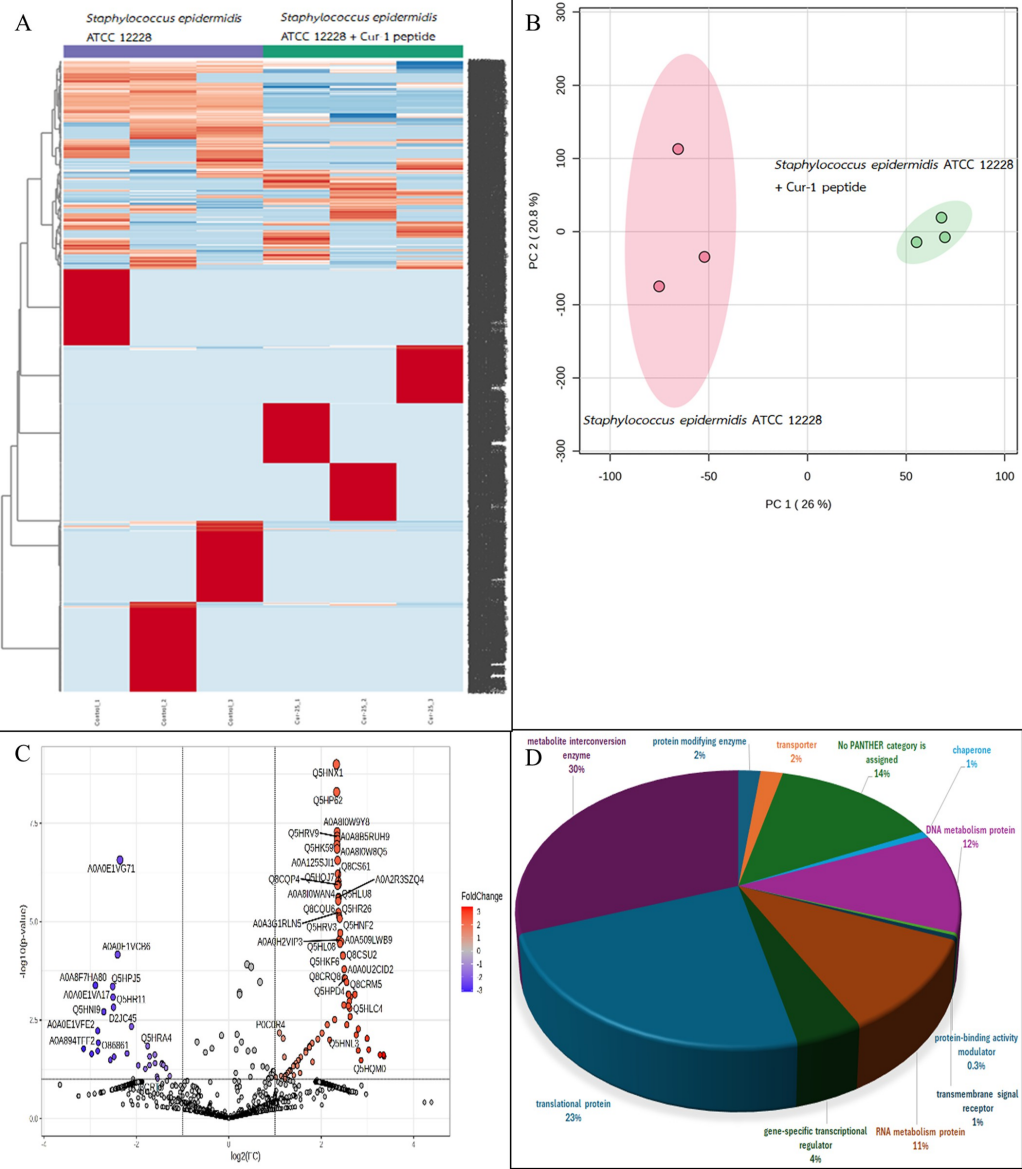
Proteomic profile of *Staphylococcus epidermidis* ATCC 12228 in response to peptide cur-1. (A) Heatmap represents protein expression level. (B) Principal component analysis of protein profiles. (C) Volcano plot analysis shows the differential expressed proteins. (D) Functional analysis of the differential expressed proteins.

A total of 2122, 1343, 2605 and 6680 proteins were identified in *A*. *baumannii*, *S*. *aureus*, *S*. *epidermidis*, and *S*. *enterica* after exposure to Cur-1 peptide, respectively (Figs 1A–4A). Principal component analysis (PCA) showed moderate separation of the bacterial proteins in response to Cur-1 as shown in Figs 1B–4B. A volcano plot with subsequent Tukey’s *post hoc* analysis, revealed significant differences (*p* < 0.05) in 85 out of 2122, 27 out of 1343, 117 out of 2605 and 109 out of 6680 identified proteins in *A*. *baumannii*, *S*. *aureus*, *S*. *epidermidis*, and *S*. *enterica* after exposure to Cur-1 peptide, respectively (Figs 1C–4C). The results showed the expression of several proteins in all bacteria implicated in a variety of biological functions (Figs 1D–4D)

### Antioxidant activity of synthetic peptides derived from turmeric hydrolysate

The antioxidant activities of synthetic peptides derived from turmeric hydrolysate were evaluated using ABTS, DPPH and FRAP assay. The results varied according to the assay used. As shown in Table 6, the synthetic peptides Cur-1 to Cur-14 demonstrated ABTS antioxidant activity with values ranging from 0.00 to 0.30 μg AAE g^−1^. Antioxidant activity against DPPH of peptides Cur-1 to Cur-14 fell within the range of 0.52–0.77 μg AAE g^−1^ while displayed FRAP values ranged between 0.90 and 1.08 μg μg AAE g^−1^.

**Table 6:** ABTS radical-scavenging activity, DPPH radical scavenging activity, and FRAP of turmeric crude hydrolysate, bound, and synthetic peptide.

Sample	Antioxidant activities (μg AAE g^−1^± SD)		
	ABTS	DPPH	FRAP
Cur-1	0.22±0.00	0.63±0.04	1.05±0.01
Cur-2	0.13±0.00	0.75±0.03	1.05±0.01
Cur-3	0.08±0.00	0.67±0.00	1.04±0.00
Cur-4	0.15±0.00	0.77±0.01	1.08±0.09
Cur-5	0.12±0.00	0.62±0.01	1.04±0.00
Cur-6	0.09±0.01	0.67±0.06	1.05±0.00
Cur-7	0.15±0.00	0.74±0.02	1.04±0.00
Cur-8	0.13±0.00	0.58±0.00	1.05±0.01
Cur-9	0.07±0.00	0.70±0.00	1.04±0.02
Cur-10	0.30±0.01	0.75±0.04	1.04±0.00
Cur-11	0.17±0.01	0.68±0.02	1.05±0.01
Cur-12	0.02±0.00	0.60±0.06	1.04±0.01

## Discussion

Food-derived bioactive peptides are recognized as valuable components in functional foods and nutraceuticals. Turmeric (*Curcuma longa* L.) has a longstanding history as an alternative herbal remedy in Southeast Asia and India for decades. Upon ingestion, these peptides must resist the enzymatic activity encountered during their passage through the gastrointestinal tract. To release bioactive peptides during gastrointestinal digestion, the total protein isolated from turmeric was subsequently digested with pepsin under acidic conditions, simulating the gastrointestinal environment. The size of these peptides is a critical factor influencing the efficacy of antibacterial agents [24] and their ability to traverse the intestinal epithelial barrier to reach the target organs. The digested peptides with a size smaller than 3 kDa were collected.

The antibacterial activity of protein hydrolysate containing <3 kDa peptides from turmeric sample was tested by broth dilution method. These small peptides exhibited strong inhibitory activity against gram positive (*S*. *epidermidis* and *S*. *aureus*) and gram negative bacteria (*A*. *baumannii* and *S*. *enterica*). Interestingly, these small peptides could inhibit the growth of *A*. *baumannii*, *S*. *epidermidis*, *S*. *aureus* higher than ampicillin (Table 1). The cationic antibacterial peptide caP4 (2.97 kDa), isolated from *Curcuma pseudomontana* L. (Zingiberaceae), demonstrated potent antibacterial activity against *Escherichia coli* and *Staphylococcus aureus* [25]. Additionally, Raj et al. [26] reported that AMP from *Zingiber zerumbet* (plant in the Zingiberaceae family, which is the same family as turmeric) rhizomes had an inhibitory effect on *Pythium myriotylum* zoospore viability.

In addition, the turmeric protein hydrolysate possessed DPPH and ABTS radical-scavenging activity, as well as FRAP. At the tested concentration of 100 μg/mL, the <3 kDa peptides derived from turmeric hydrolysate effectively scavenged ABTS and DPPH free radicals, yielding 1.24±0.01 and 0.14±0.03 μg AAE·g^−1^, respectively. However, no FRAP reducing power was observed. After evaluation for antibacterial and antioxidant activity, the <3 kDa bioactive peptides were purified by a series of purification methods. After cation exchange chromatography, bound fractions exhibited both antibacterial and antioxidant activity. When anionic peptides were removed by anion exchange chromatography, bound fractions showed higher antibacterial activity but slightly lower antioxidant activity (ABTS and DPPH). All hydrophobic peptides bound to C18 column were stepwise eluted with acetonitrile. Peptides eluted at 60% acetonitrile exhibited both potent antibacterial and antioxidant activity. Based on these results, this fraction was selected for peptide identification by LC-MS.

Structural characteristics and physicochemical properties such as size, net charge, hydrophobicity, amphipathicity and solubility may give an insight into the mechanism of antibacterial activities [27]. The length of the peptide with at least 7–22 amino acid residues are idealistic to form α or β-sheet structures that can obstruct the bacterial membrane [28]. Furthermore, the hydrophobicity is also essential character of AMPs. Peptides comprising of hydrophobic residues are a required quality for AMPs to puncture the bacterial membrane [29].

The chemical and physical properties of all twelve synthetic peptides, named Cur1–Cur12, can be seen in Table 4. The length of synthetic peptides was 8–15 amino acids. The net-positive charge was from -0.9 to +2. The hydrophobicity of Cur-12 was the lowest (25.56%) and the hydrophobicity of Cur-1 –Cur-11 was 33.11–41.97%. Cur-3 and Cur-10 had the highest hydrophobicity at 41%. Antibacterial activity of all designed peptides was determined against four bacterial strains (*S*. *aureus* ATCC 25923, *S*. *epidermidis* ATCC 12228, *A*. *baumannii* ATCC 19606 and *S*. *enterica* ATCC 13311) as can be seen in Table 5. The peptides Cur-1 and Cur-2 at 100 μg/ml had better antibacterial activity than the other peptides. The other peptides could not inhibit growth of all tested bacteria. This was possibly caused by the defense activation of bacteria against the peptides or peptide aggregation [30]

Cur-1 and Cur-2 peptides had the same number of amino acid residue which was 8. They had similar hydrophobicity 38% and net charge of 1. It indicates that a low hydrophobicity and low net charge may result in good antibacterial activity. However, low pI and low helix structure increased their antibacterial activity [31]. In addition, Cur-3, Cur-5 and Cur-6 containing higher number of amino acid residue, had poor antibacterial activity against all bacterial strains indicating that a longer peptide chain and a more positive charge do not improve antibacterial activity.

However, the peptide Cur-1 (KLHLLILI) presented the highest inhibitory activity against *A*. *baumannii*. The possession of 4 Leucine, 1 Histidine, 2 Isoleucine and 1 Lysine at the N-terminal in Cur-1 justified hydrophobic nature and the amphipathicity of the peptides accretes membrane permeability of bacterial cells. The cationic amino acid residues are shown to interact with negatively charged phospholipids of the bacterial cell membrane [32,33]. The synthetic peptides with a net charge of 1, possibly one of the factors contributing to antibacterial activity against *A*. *baumannii*, *S*. *aureus*, *S*. *epidermidis*, and *S*. *enterica*. Another important factor contributing for antibacterial activity of the peptides is the hydrophobicity [34,35], net charge, pI and helix structure in Cur-1 peptide has led to explore the possible mechanism of antibacterial activity.

To attempt to understand how Cur-1 peptide exerts its anti-bacterial effect, label-free shotgun proteomic approach was utilized. *A*. *baumannii*, *S*. *aureus*, *S*. *epidermidis*, and *S*. *enterica* were therefore treated with 100 μg/mL Cur-1 peptide and at 24 h post treatment, cells were harvested, and total proteins prepared, reduced, alkylated, trypsin digested and subjected to LC-MS. Expression level of 85, 27, 117 and 109 proteins identified in *A*. *baumannii*, *S*. *aureus*, *S*. *epidermidis*, and *S*. *enterica* was significantly changed in their abundance after exposure to Cur-1 peptide, respectively. Proteins playing role in metabolite interconversion were mainly increased in *A*. *baumannii*, *S*. *epidermidis*, and *S*. *enterica* while expression level of translation related proteins were significantly changing in *S*. *aureus*. This effect is similar to some previous observations. Three synthetic peptides from bagasse inhibit growth of three pathogens: *Pseudomonas aeruginosa*, *Bacillus subtilis*, and *Burkholderia cepacian* by intracellular active mechanism [36].

Antioxidants are known to propose safeguarding to cellular macromolecules against oxidative injury. They can either extinguish or scavenge reactive oxygen species (ROS), or act as chain-breaking antioxidants, by being converted into nonradical or weakly oxidant products during the reactions [37]. Many peptides with antioxidant activity have been isolated from hydrolysates. The short peptides obtained from casein and lactalbumin hydrolysates had good antioxidant activity [38,39]. Srinivas et al. [40] reported that a water-soluble antioxidant peptide (turmerin) isolated from turmeric possessed efficient antioxidant. In this study, all 12 synthetic peptides derived from turmeric proteins exhibited antioxidant activity. Cur-10 demonstrated the best antioxidant activity among the synthetic peptides. A comparison between Cur-1, Cur-2, Cur-4, Cur-7, Cur-8, Cur-10 and Cur-11 which contain 8 amino acid residues with different hydrophilicity (33.11–41.97%) and net charge (0.1–2) showed that antioxidant activity depends on net charge, pI and hydrophobicity.

## Conclusions

Turmeric can be characterized as a source of antimicrobial and antioxidant peptides. Protein hydrolysates isolated from turmeric exhibited antibacterial activity against human bacterial pathogens. Peptides Cur-1 (KLHLLILI) was identified as novel peptides derived from turmeric that inhibited the growth of *A*. *baumannii*, *S*. *aureus*, *S*. *epidermidis*, and *S*. *enterica*. The antibacterial activity of this peptide is through affecting proteins playing role in metabolite interconversion and translation. Cur-1 exhibited antioxidant activity for ABTS, DPPH and FRAP. Not only the hydrophobicity, net charge and pI but also helix structure are important factors for antibacterial and antioxidant activity of peptide. Cur-1 could be potentially AMPs and antioxidant peptides as food industrial applications proffering a novel approach for the control of pathogenic bacteria.
